# Choroidal vascularization and adrenergic innervation qualitative findings obtained with induced fluorescence preparations and optical coherence tomography angiography: possible correlations and perspectives

**DOI:** 10.1186/s40942-020-00255-8

**Published:** 2020-11-30

**Authors:** Raffaele Nuzzi, Federico Tridico, Alessandro Marchese, Francesco Bandello

**Affiliations:** 1 Ophthalmology Unit, A.O.U. City of Health and Science of Turin, Department of Surgical Sciences, University of Turin, Via Cherasco 23, 10100 Turin, Italy; 2 Department of Ophthalmology, San Raffaele Scientific Institute, University Vita-Salute San Raffaele, Via Olgettina 60, 20132 Milan, Italy

**Keywords:** Choroid, Optical coherence tomography, OCT angiography, Immunohistochemistry, Adrenergic innervation

## Abstract

**Background:**

Recent advances in optical coherence tomography (OCT) technology allow a more accurate choroidal visualization. The aim of this study is to provide histochemical analysis with induced fluorescence images of the choroidal stromal, vascular and nerve network, highlighting possible correspondences with OCT and OCT angiography (OCT-A) analysis.

**Methods:**

The material examined with a histochemical process of induced fluorescence through condensation of biologically active monoamines with glyoxylic acid was obtained from 6 eyes enucleated for malignant melanoma and ciliary body neoplasia. The resulting images have been qualitatively compared with OCT and OCT-A choroidal images obtained from 10 volunteers, in order to identify possible relationships. Choriocapillary segmentation was performed automatically through the embedded analysis software, while segmentation of Sattler’s and Haller’s layers was performed through a manual method.

**Results:**

Histochemical analysis provided accurate visualization of choroidal adrenergic innervation across all layers and its relationships with blood vessels and melanocytes. The above structures were not visualized at OCT and OCT-A which provided good visualization of blood vessels in Sattler’s and Haller’s layers as well-delimited hyporeflective areas. Decorrelation signal was not detected in OCT-A analysis due to low blood flow velocity in external choroidal layers.

**Conclusions:**

The choroid is an extremely dynamic structure which deserves to be analyzed in vivo since it is involved in the pathogenesis of several ocular conditions. Direct evaluation of the activity of choroidal nerves and melanocytes is still not possible with OCT and OCT-A, even if they are capable of providing a satisfactory representation of choroidal vascularization.

## Background

The choroid is the largest vascular network of the eye. Besides being rich of blood vessels, it presents a large stroma of interlaced connective tissue, melanocytes and nerve fibers [1]. Choroidal innervation has been studied with several methods, including histochemical techniques. Among these, induced immunofluorescence proved to be the most reliable and reproducible [1–3]. The choroid is innervated by short and long posterior ciliary nerves, which supply the choroidal amyelinated fibers and form an extended plexus [4]. Nerve fibers run along the muscular wall of choroidal arteries. Large nerves follow large vessels and free terminations are localized into arterial walls. Paravenous nerves are also present, but in a lower frequency. An additional nerve network is located adjacent to the Bruch membrane.

Recent advances in optical coherence tomography (OCT) technology have revolutionized both research and clinical practice in chorioretinal disorders. Several OCT techniques can be used to better visualize the choroid: enhanced-depth OCT imaging (EDI-OCT), swept-source OCT (SS-OCT) and image averaging. One of the most recent developments led to OCT angiography (OCT-A), which can reliably document retinal and choroidal vascularity, in a non-invasive and reproducible manner [5–11].

The aim of this study is to provide histochemical analysis through induced fluorescence of the choroidal stromal, vascular and nerve network, highlighting possible correspondences between histological images and those observed on OCT and OCT-A.

## Methods

Histological preparations were examined with a histochemical technique of induced fluorescence (IF), according to the method described by Furness and Costa [12], which exploits the condensation of biologically active monoamines with glyoxylic acid to obtain fluorophores with detailed localization and more intense fluorescence when compared with formaldehyde-based condensation methods, with reduced risk of artifacts and changes during preparation, since it is faster and easier. Moreover, this induced fluorescence technique provides better resolution and higher fluorescence yield if compared to the Falck–Hillarp method, especially in extensive preparations [12].

The examined material was obtained from 6 eyes enucleated for malignant melanoma and ciliary body neoplasia. Patient characteristics are listed in Table 1. All lesions were localized in lower temporal sectors. The choroidal collection, carried out at a distance from the lesion, included upper and lower nasal sectors and the upper temporal sector. The excised area extended posteriorly to include the optic nerve and posterior pole and anteriorly till the retinal equator. The excided tissues (including sclera, choroid, and retina) were immersed in a 2% glyoxyl-phosphate solution for no more than 2 h. With the aid of a dissecting microscope, an accurate separation of sclera and retina was carried out through tangential traction. The obtained choroid preparation was introduced again in the same glyoxylic phosphate solution. Afterward, three layers were separated from each choroidal sample:

- The large vessels layer with residues of the supra-choroidal lamina.
- The medium-sized vessels layer.
- A final layer composed of choriocapillary and Bruch's membrane.

**Table 1:** Enucleated patients’ characteristics

***Enucleated patients’ characteristics***	
***Sex***	
Male (N)	2
Male (%)	33.33%
Female (N)	4
Female (%)	66.67%
***Age***	
Range	54–63
Average	61.5
***Cases of choroidal melanoma***	N = 5
	83.33%
***Cases of ciliary body neoplasia***	N = 1
	16.67%
***Total***	N = 6

Each layer was then spread on glazing slides until dehydration at room temperature for about 5–10 min. The preparation was placed in a dry oven at 100 °C for 4 min, in order to obtain the fluorophore by condensation of the intracellular catecholamines with the glyoxylic acid.

Microscopic sample observation was performed using a fluorescence microscope, equipped with two filters for excitation and emission wavelengths (specific for catecholamines and indoleamines).

The corresponding fluorescence images were then acquired and compared with OCT (b-scan and “en face”) and OCT-A choroidal images from 10 volunteers, in order to identify possible relationships (Cirrus 5000 AngioPlex, Carl Zeiss Meditec, Inc., Dublin, USA). Choriocapillary segmentation was performed automatically through the embedded analysis software. On the other hand, Sattler and Haller layers segmentation was performed manually by setting up custom slabs. Given the considerable variability of choroidal thickness between individuals, manual segmentation has been performed by the examiner through a qualitative and customized method for each individual patient, in accordance with the following graphic cut rules:

- Sattler layer: slab including medium-sized vessels immediately below the choriocapillary up to the interface with the larger-sized vessels.
- Haller layer: slab extending from the transition zone of the previous layer up to the sclero-choroidal interface.

Given the complex deep choroid visualization due to retinal pigment epithelium (RPE) reflective properties, 3 individuals suffering from pathological myopia or geographic atrophy, affected by retinal and RPE atrophy at the posterior pole were included in the sample under study.

This study has been conducted according to the ethical standards of our Institution and in conformity with the principles set out in the Declaration of Helsinki and its revisions. Consent to participate has been collected in written form for all the subjects under study.

## Results

### Fluorescence histological preparations

**Fig. 1:**
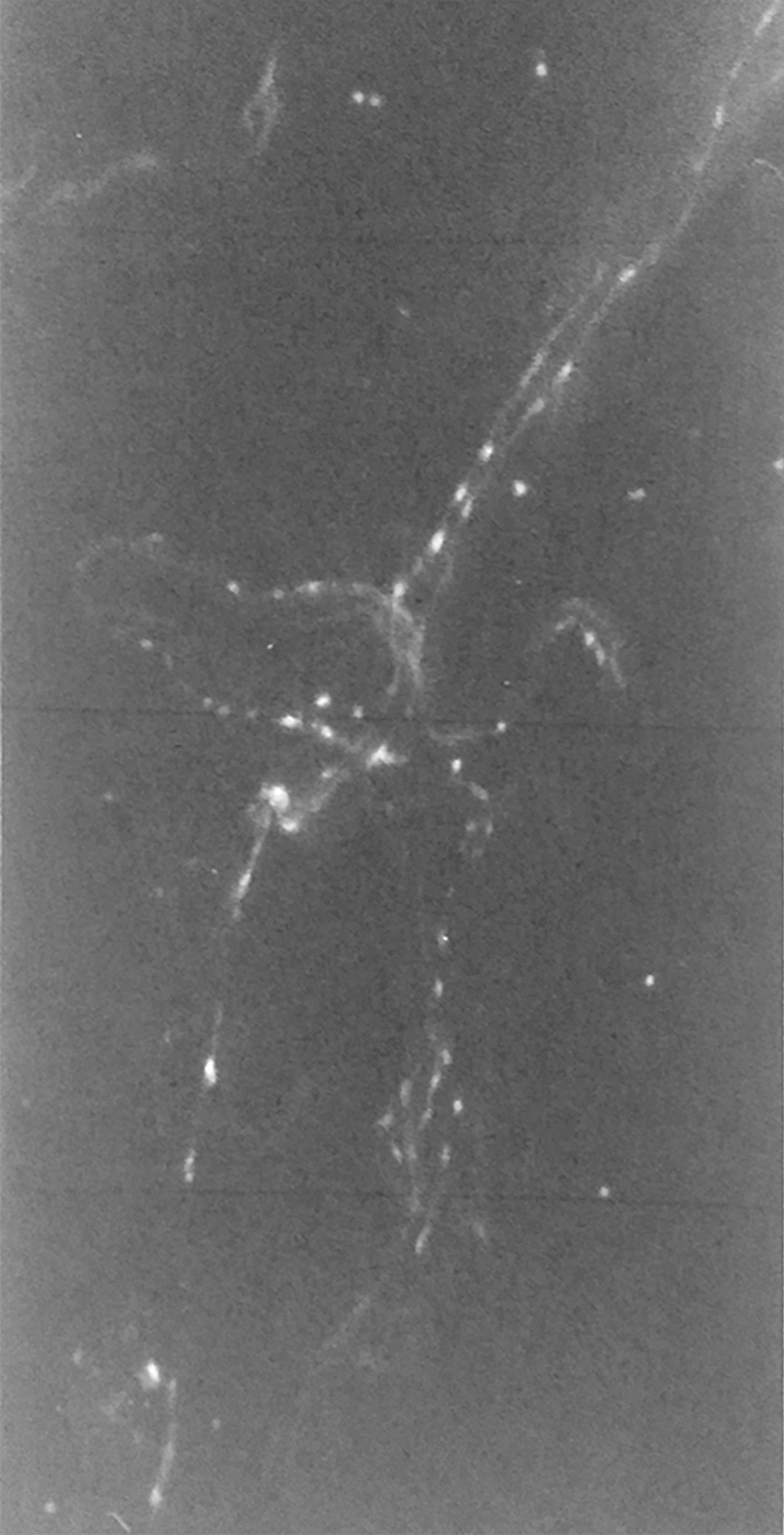
Histologic observation of ciliary nerve branches with poor fluorescence at the level of the suprachoroidal lamina (×400)

In the preparations regarding the suprachoroidal lamina, we found the ciliary nerves, with a rectilinear course, directed towards the ciliary body. The branches that these nerves yield to the underlying vascular lamina are preserved intact. Among the choroidal branches of posterior short ciliary nerves, there is a certain number of fibers with specific fluorescence for catecholamines. From the ciliary branches, there were numerous collateral bundles consisting of intensely fluorescent fibers whether of tortuous or linear distribution. Only a small part of the fibers originating from ciliary nerves branches appear fluorescent at the level of the suprachoroidal lamina (Fig. 1).

A large number of postganglionic adrenergic orthosympathetic fibers reach the choroid by passing in perivascular nerve pathways or in the context of the periarterial plexus (with a linear course and moderate fluorescence). Fibers forming the nerve network in the context of the arterial adventitia tunic feature a varicose and intensely fluorescent appearance, hardly distinguishable from the terminal fibers directed to vascular smooth muscle fibers.

**Fig. 2:**
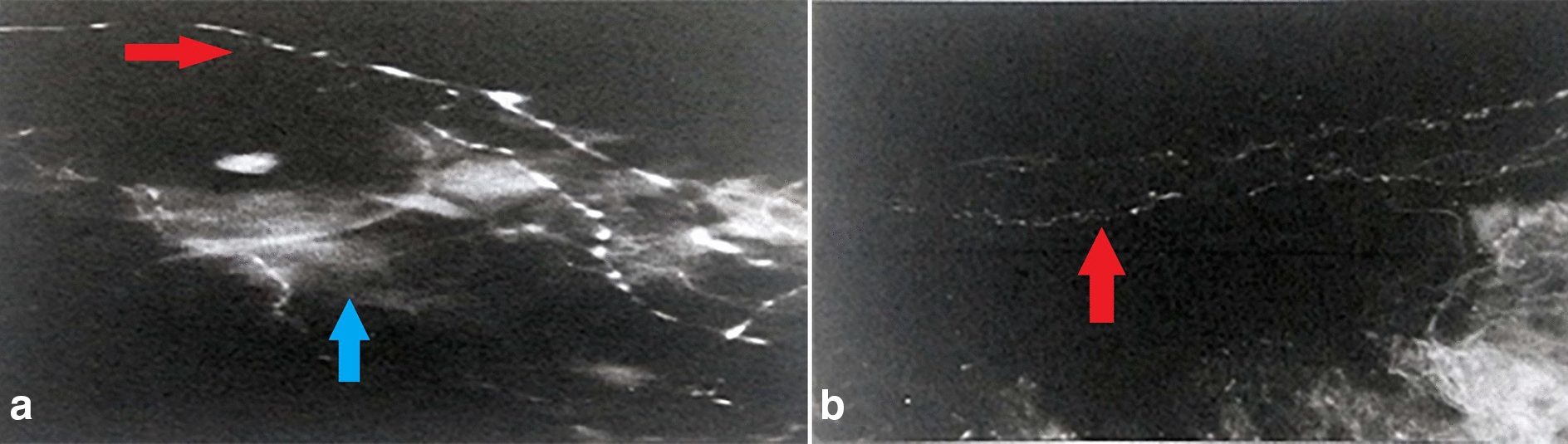
**a** Para-arterial neural branches with adrenergic fibers (blue arrow) featuring relationships with the vasal smooth muscle (red arrow). **b** Adrenergic fibers distribution within the arteriolar wall (×500)

**Fig. 3:**
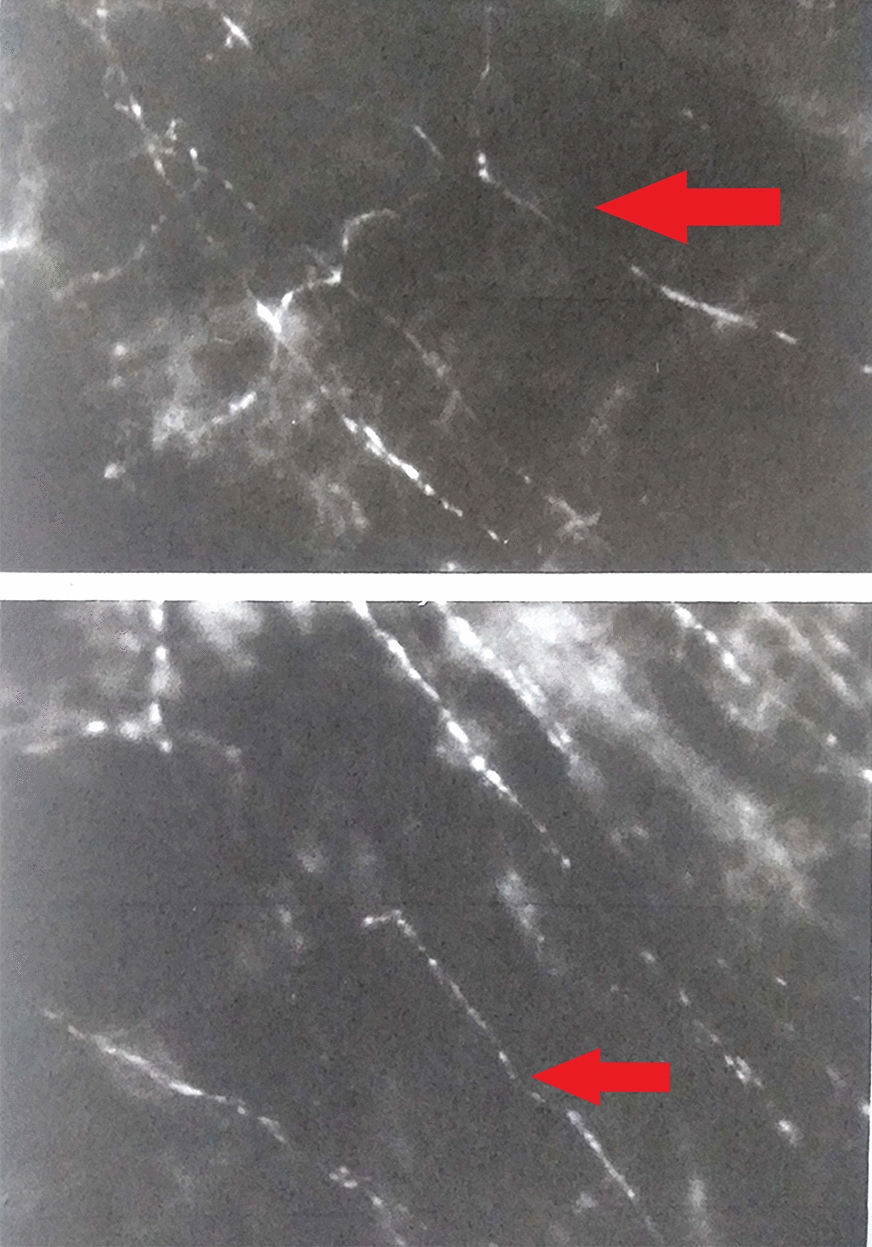
Perivascular terminal adrenergic fibers with an independent network in relation with blood vessels (×800, blue arrow)

**Fig. 4:**
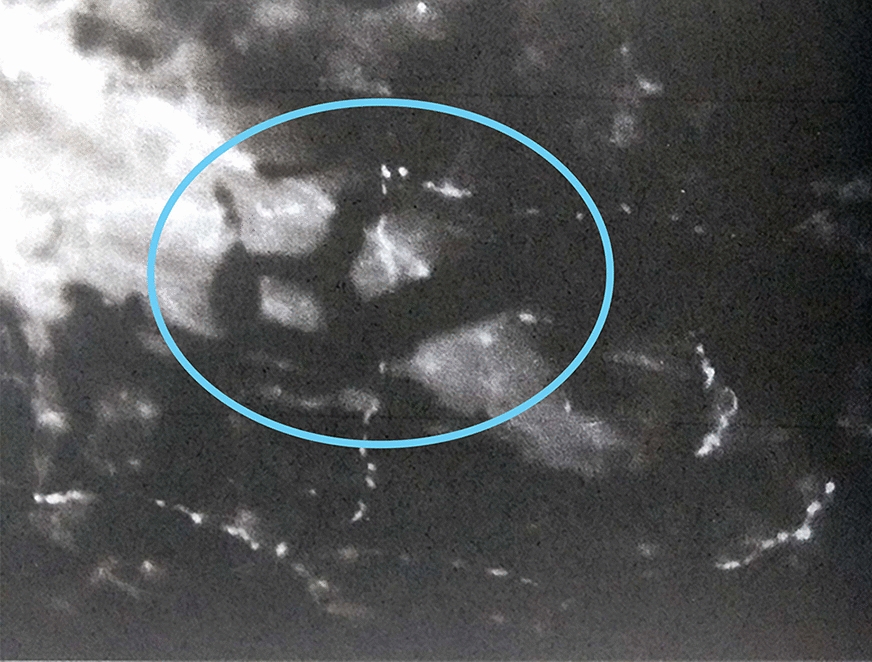
Adrenergic terminal fibers intertwining with melanocytes’ pedicles

Arterial vessels of high and medium caliber are surrounded by bundles of preterminal nerve fibers, whose branches, at the level of the vascular lamina, intertwine with those derived from collateral branches of the ciliary nerves, forming a dense network of terminal adrenergic fibers (Fig. 2). At the level of Haller’s layer, spread small intensely fluorescent cells (SIF cells), with neuroendocrine function, were identified. With the progressive reduction of the vessel caliber, the periarterial neural plexi become independent from vascular vessels and flow into an intervascular terminal network (Fig. 3). This network is particularly developed in the posterior sector of the deepest section of the vascular lamina and features moderately fluorescent preterminal fibers or fibers with intensely fluorescent terminal varicosities. In the anterior part of the vascular lamina, these fibers tend to be rarer and run within intervascular spaces of smaller and smaller width until they form an extremely thin terminal arborization. In the observed fluorescence preparations, intervascular spaces are occupied by numerous melanocytes that appear as intensely dark cells in contrast to the aspecific choroidal background fluorescence and contract relationships with the choroidal adrenergic innervation. In fact, the fluorescent network of terminal adrenergic fibers overlaps with the cytoplasmic (non-fluorescent) melanocytes extensions next to large vessels of the superficial layer of the vascular lamina. At the level of the intervascular spaces, fluorescent fibers cross the cytoplasmic melanocytic prolongations and contact melanocytes’ cell body through intensely fluorescent synapses, suggesting a direct melanocytic innervation by orthosympathetic nerve fibers (Fig. 4).

A group of neurons with non-specific autofluorescent granular content has been observed at the suprachoroidal level. These neurons can be distributed along the course of ciliary nerve branches. Alternatively, they do not have any apparent relationship, maintaining, however, a relationship with contiguous nerve fibers with catecholamine-specific fluorescence.

Similar to the arteries, even the highest caliber veins located at the supra-choroidal lamina or in the posterior section of the vascular lamina possess varicose adrenergic fibers, which form a wide-mesh fluorescent network interesting the adventitia tunic. However, the venous adrenergic endowment is exclusively limited to the large diameter vessels and it’s less represented when compared with the arterial one, with irregular and intensely fluorescent varicosities.

At the level of the vascular lamina, the nerve branches originating from the ciliary nerves exchange bundles of fluorescent fibers, forming an irregular meshwork with significantly large varicosities, overlapped to the overlying layer of larger diameter vessels at the level of the borders of the suprachoroidal lamina. Moreover, in the histochemical analysis of the choriocapillaris, it was not possible to observe fluorescence for catecholamines, related to adrenergic fibers.

### OCT images

**Fig. 5:**
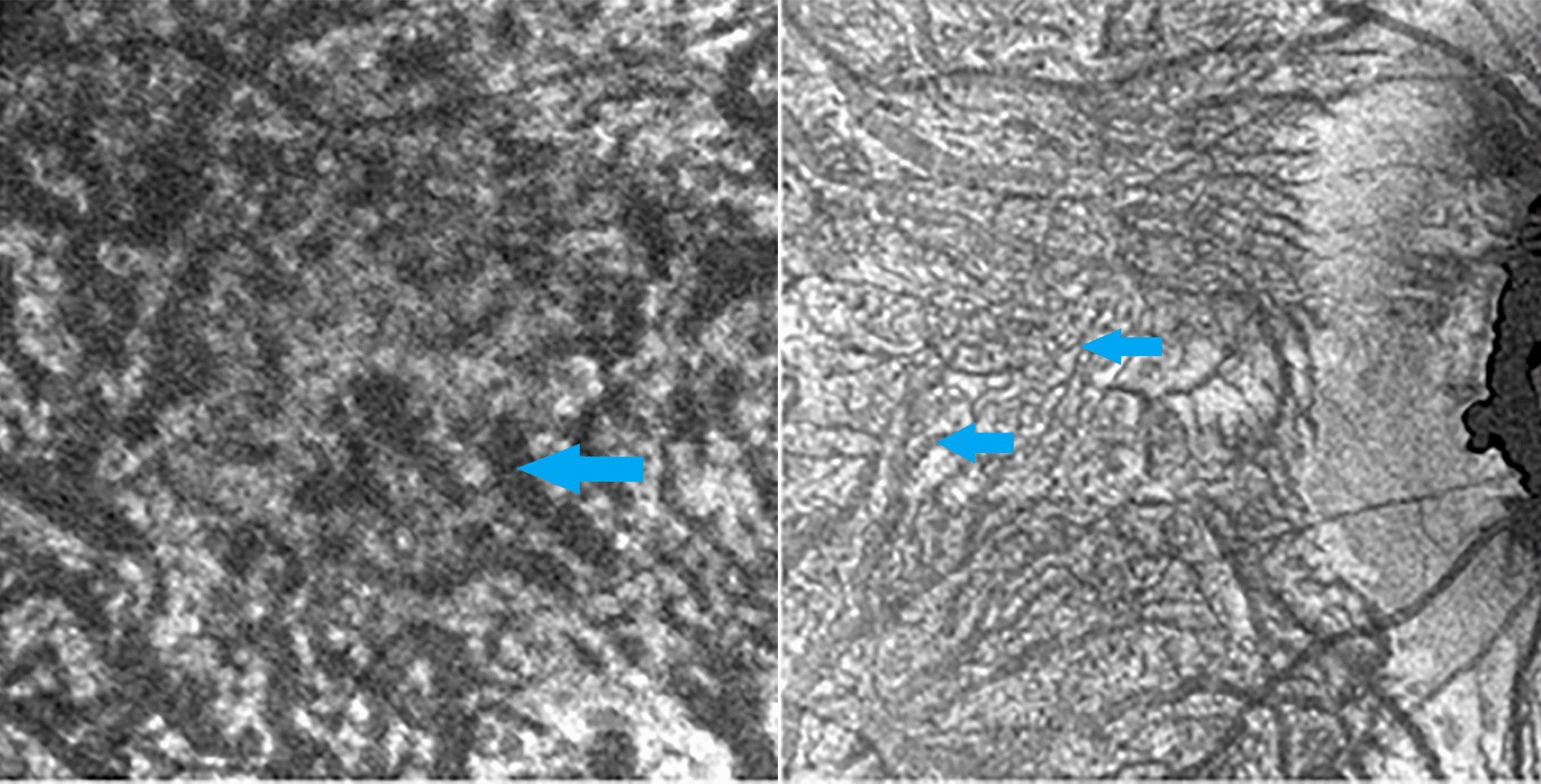
“en face” OCT analysis of Haller layer (OS, image on the left) and Sattler layer in a myopic patient (OD, image on the right). Sattler layer was not detectable in patients with regular fundus due to reflective properties of healthy EPR. Large vessels are represented by hyporeflective areas in the surrounding stroma (blue arrow)

OCT B-scan and C-scan “en-face” images of three choroidal layers (choriocapillary, Sattler or intermediate layer, Haller or external layer) were acquired. It was possible to visualize the choroidal vascularization at the level of the Sattler and Haller layers as hypo-reflecting curvilinear areas—well defined by the surrounding hyper-reflective stroma—both in B-scan and “en face” scans (Fig. 5).

### OCT angiography

**Fig. 6:**
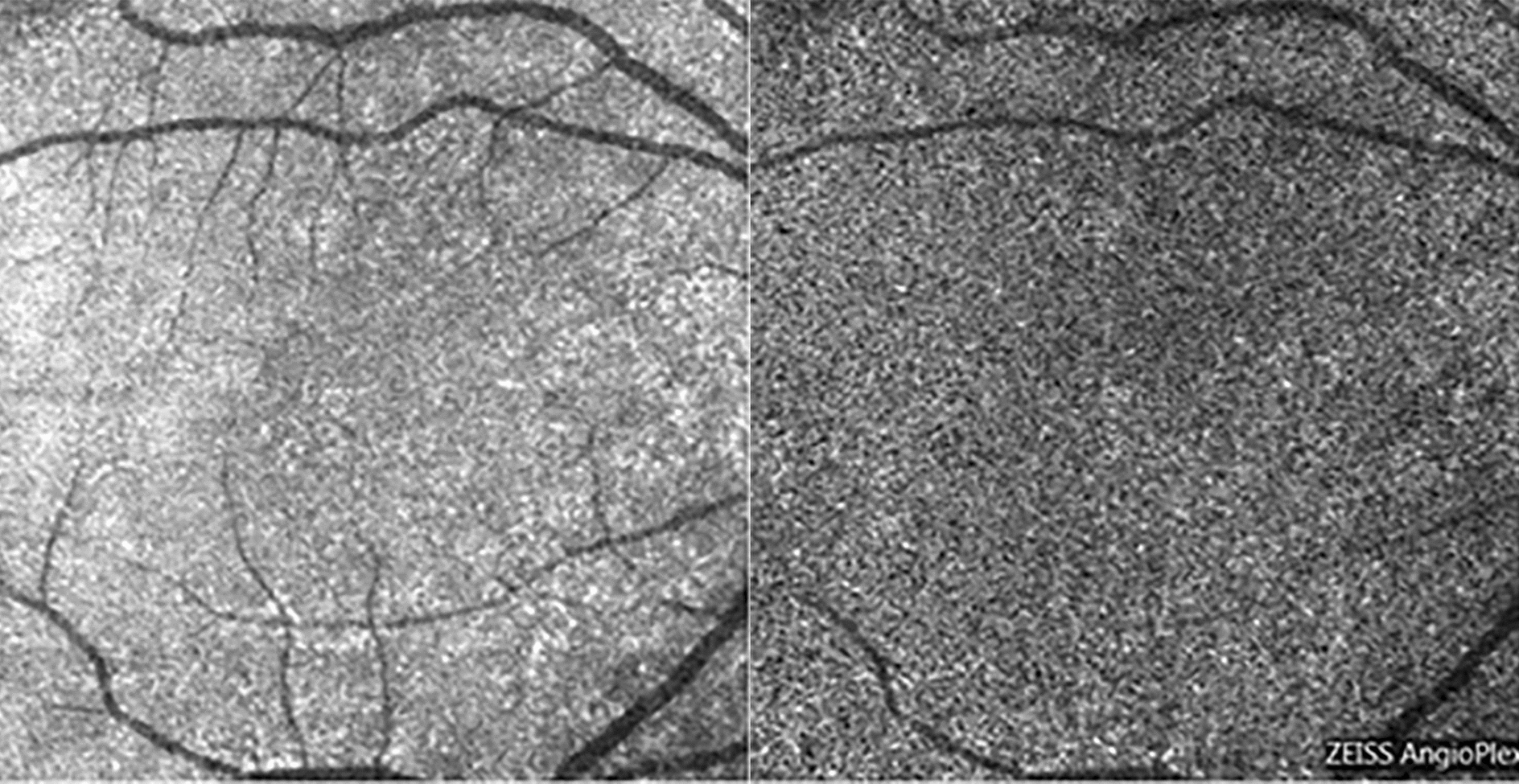
Choriocapillaris in OCT (image on the left) and OCT-A “en face” analysis (image on the right)

**Fig. 7:**
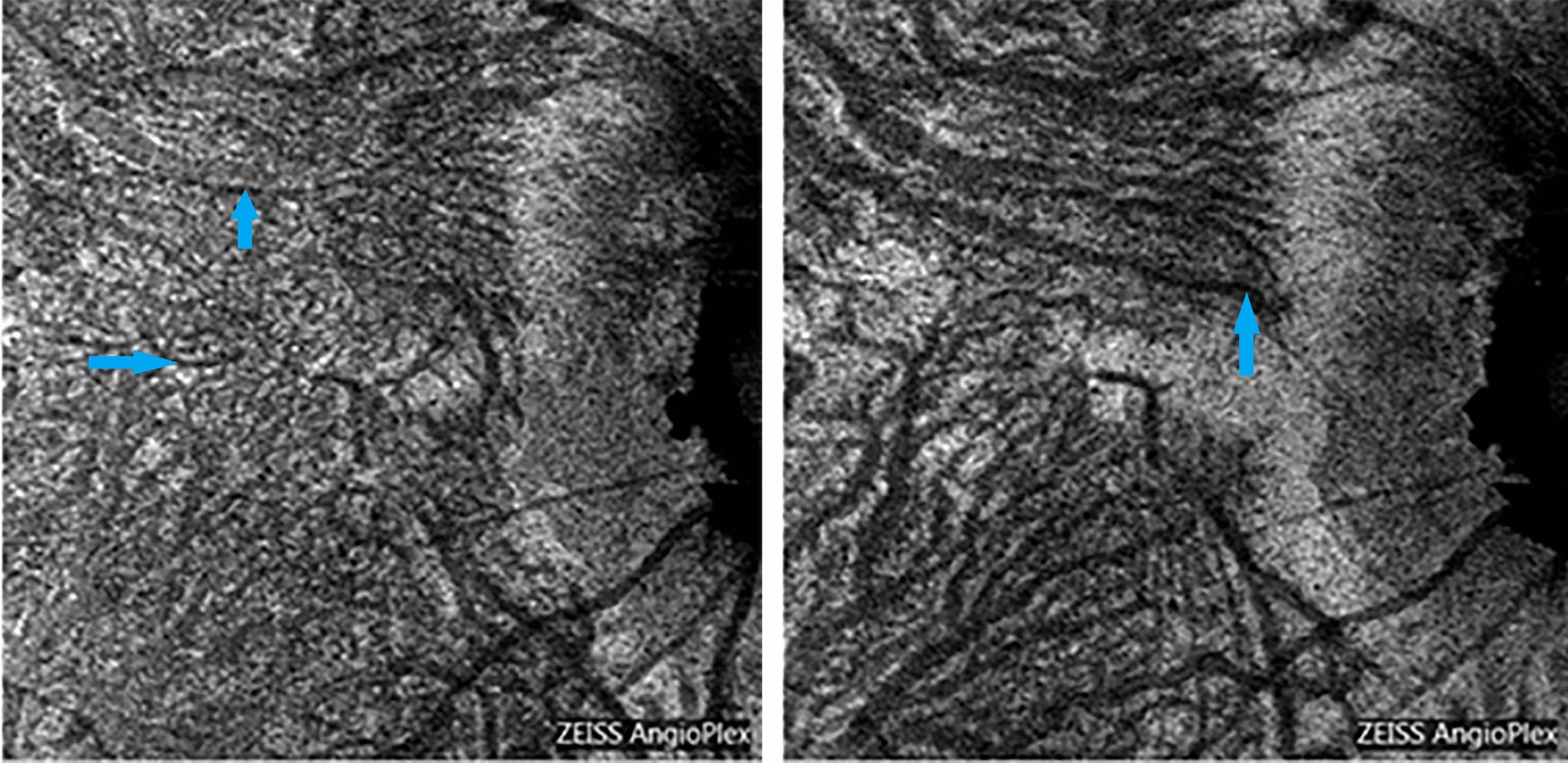
Sattler (image on the left) and Hattler (image on the right) layers in a myopic patient visualized in OCT-A “en face” analysis. Choroidal vessels are visualized as hyporeflective areas (blue arrow)

The same layers have been studied with OCT angiographic method, with “en face” scans. The anatomical structure of the choriocapillary is not clearly displayed, but the resulting image is characterized by a granular appearance of light and dark areas of different sizes and the individual capillaries are not clearly visible (Fig. 6). The flow in the deeper layers of the choroid cannot be visualized at OCT A, due to the reflective properties of the retinal pigment epithelium, and appeared as darker areas corresponding the vascularization of the Sattler and Haller layers (Fig. 7).

### Histological findings and OCT images compared

**Fig. 8:**
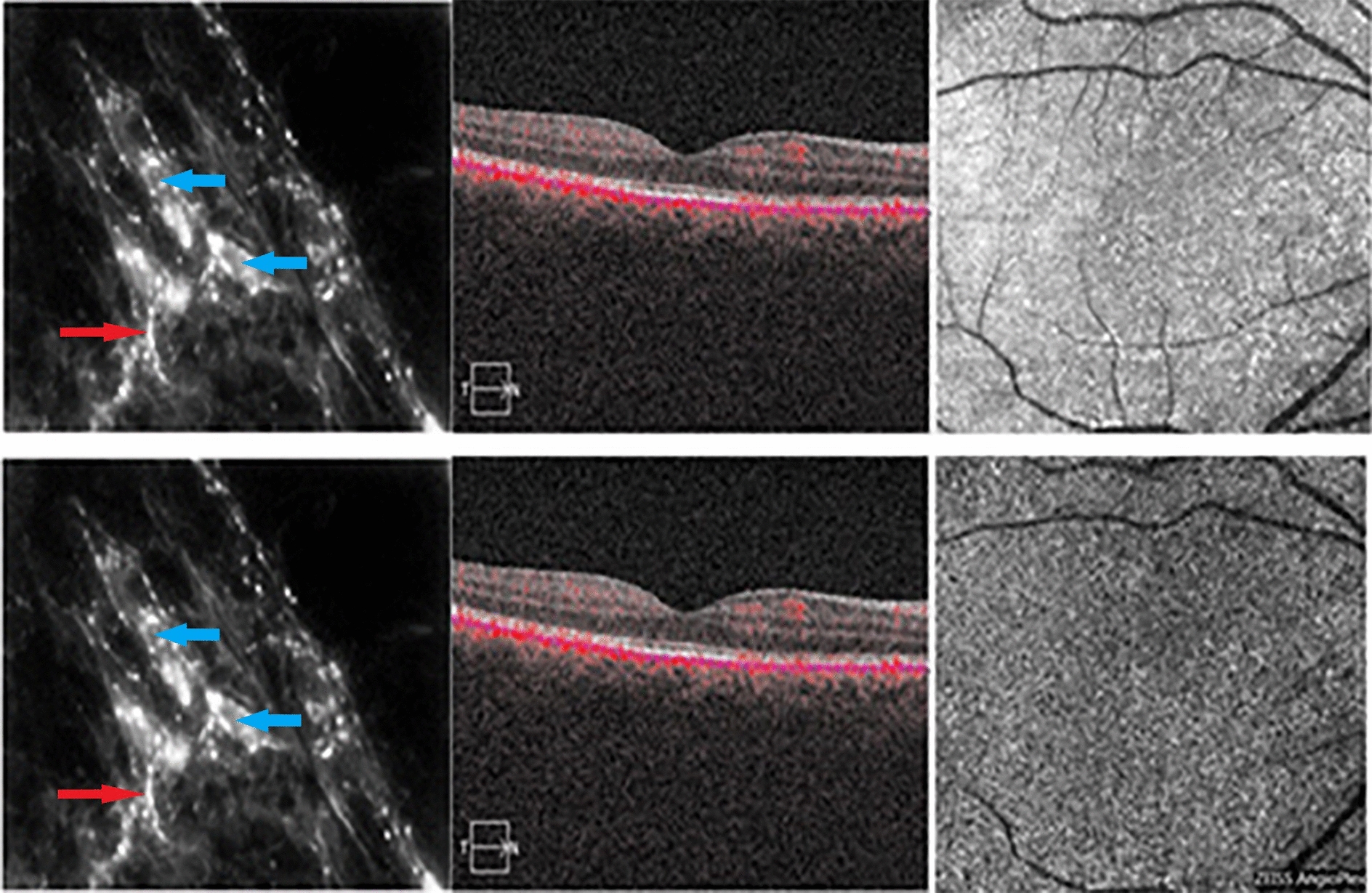
Intervascular adrenergic nerve fibers (red arrow) in contact with melanocytes (blue arrow, images from the left) and visualization of choriocapillary layer in B-Scan OCT + OCT-A (images in the middle) and “en face” OCT (upper-right image) and OCT-A (lower-right image). The Sattler layer is not clearly visible in OCT-A due to the reflective properties of retinal pigment epithelium

The perivascular and intravascular adrenergic innervation (hyperfluorescent in histological images), which contracts relationship with the melanocytes at the level of the choroidal vascular lamina, have not been identified in the OCT-B scan images, in which the stroma of the different layers appears uniformly hyper-reflective. The vascularization in the different layers is visualized as intensely black areas well delimited both in the OCT-B images and in the “en face” images, especially for large and medium-sized vessels. The “en face” images did not provide information on the distribution of intrastromal and perivascular nerve fibers and melanocytes, as part of the background hyperreflectivity of the choroidal stroma (Fig. 8).

### Histological findings and OCT- angiography images compared

**Fig. 9:**
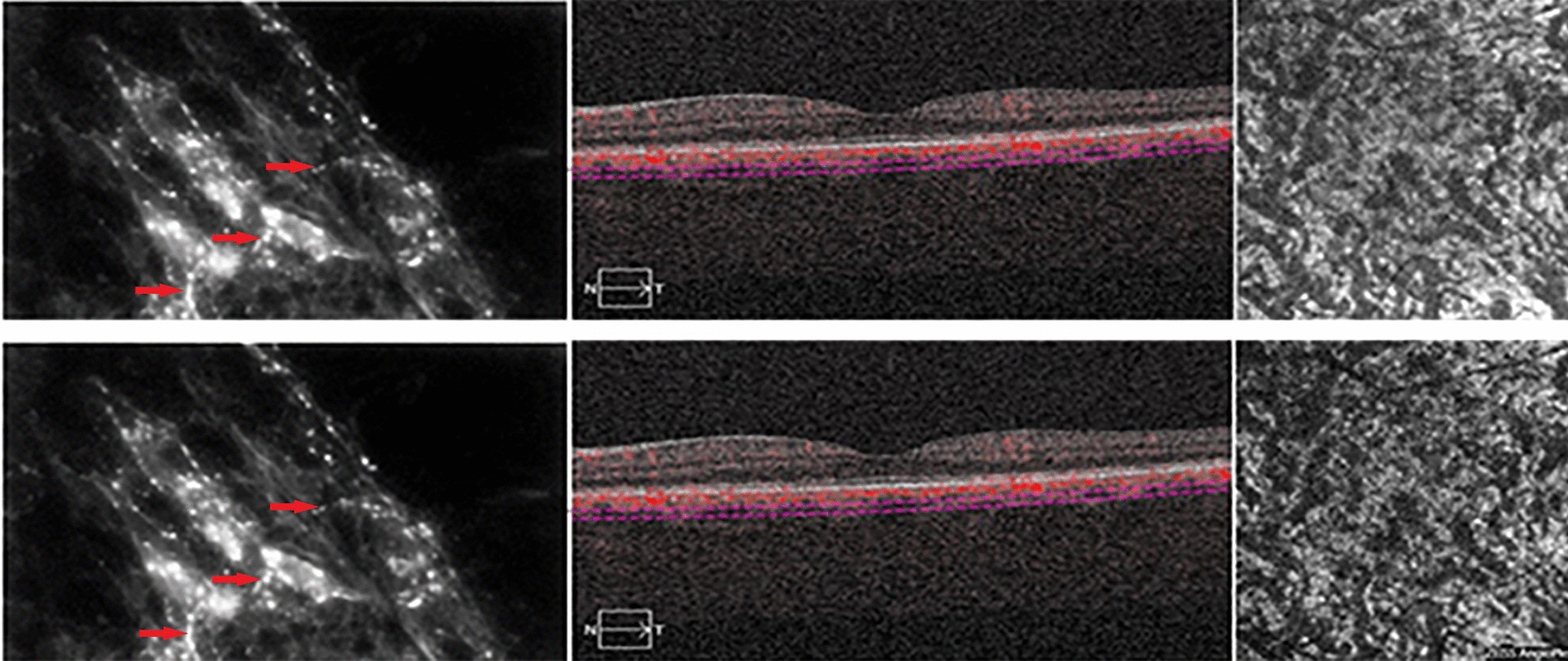
Adrenergic nerve fibers in intervascular spaces (red arrow, images from the left) and visualization of Haller layer in B-Scan OCT + OCT-A (images in the middle) and “en face” OCT (upper-right image) and OCT-A (lower-right image)

In OCT-A images, the choriocapillary layer appears to be granular, with hyper-reflective areas alternating with hyporeflective zones, without an evident delimitation of the capillaries. In some choriocapillary angiographic images, it is possible to visualize some artifacts due to the projection of the retinal vessels on the underlying choroid. Similarly to “en face” images, the stroma of the vascular lamina appears uniformly hyper-reflective, alternated with well-defined and branched hyporeflecting areas, which characterize the medium and large caliber blood vessels. The adrenergic fibers or other neural properties at the stroma level of the Sattler and Haller layers cannot be identified (Fig. 9).

## Discussion

With this study, an important regulatory system of choroidal blood flow, featuring adrenergic innervation and choroidal melanocytes, has been described through histochemical analysis. Unfortunately, choroidal elements showed in the induced fluorescence images, such as nerve fibers and melanocytes, are not visible in OCT images, due to spatial resolution limitations of OCT technology. For this reason, the information provided by these two different modalities cannot be combined. Therefore, while designing this study, it has been decided to apply the histochemical modality only to the enucleated eyes for malignant ocular disease, recognizing also that data derived from different patients cannot be put in relation to each other. Nevertheless, the aim of this study was to evaluate if the information provided by an ex-vivo modality could open novel perspectives that may be taken into consideration while approaching the study of choroidal vascularization with OCT angiography, which represents an in-vivo and noninvasive modality.

The choroid is organized into a complex plexiform network, innervated by fibers derived from the ciliary nerves. The orthosympathetic fibers, deriving from the superior cervical ganglion, reach the vascular plexi of the posterior ciliary arteries, while those deriving from the ciliary ganglion innervate the ciliary muscles and constrictor of the pupil.

The fluorescence examination of the choroid treated with glisoxyl acid allowed a precise reconstruction of the catecholaminergic fibers and their distribution in the different choroidal layers [1]. While we observed adrenergic fibers also in the deeper layers of the vascular lamina, these fibers were not detected in the choriocapillary. Regarding the venous system, only the vorticous veins and the high-diameter veins showed an adrenergic innervation. Due to this configuration, the choroidal adrenergic fibers seem to be a relevant arterial vasomotor regulating system, but it does not seem to play an important role in venous tone control. A network of intervascular adrenergic fibers has also been highlighted; whose target does not appear to be purely vascular. These nerves (identified in previous chicken models) may play a role in the control of smooth muscle cells contraction in the choroidal stroma [13]. The smooth muscle cells activity could be correlated with the regulation of intraocular pressure and the outflow of choroidal interstitial fluids.

It was also observed that adrenergic intervascular nerve fibers contract relations with the melanocytes present in the choroidal stroma. Previous studies reported the presence in rabbits of adrenergic nervous terminations in relation to choroidal melanocytes, similar to synapses, even if the functional significance of this innervation still remains obscure [14]. The contractile properties of melanocytes have been previously reported, as they possess actino-like filaments in the context of their cytoplasm [15]. This suggests a regulation of choroidal blood vessels’ caliber (especially at the level of the choriocapillary, where there are no signs of innervation) triggered/modulated by the synapses that melanocytes contract with neurons.

Thanks to advances in ophthalmic imaging technology, it is now possible to precisely delineate choroidal thickness in a reproducible manner. The most efficient algorithms allow a three-dimensional evaluation of choroidal thickness, producing volumetric maps of the total choroidal thickness measured from the retinal pigment epithelium to the sclero-choroidal interface [6, 16, 17].

In our study, OCT scans were qualitatively analyzed to identify possible relationships with the distribution of vessels and adrenergic nerve fibers identified in fluorescence histological preparations. In the B-scan and C-scan images, it was possible to determine the contour and the course of the choroidal vascularization, especially in the intermediate and outer layers of the choroid. The surrounding stroma appeared as a uniform hyper-reflective area, and the suprachoroidal lamina was not usually appreciable. Furthermore, the axial position of the Sattler and Haller layers, obtained with manual segmentation, was always approximate, as these two layers do not have a precise demarcation and so the location of their relative sections. Automated segmentation of Sattler and Haller layers is possible and reproducible, as shown in previous studies [18–22]. However, as of today, automated choroidal segmentation algorithms are expensive, hardly available on the market, and are not fully embedded in the analysis software of most widespread OCTs [22].

On OCT-A large-sized vessels appeared hypo-reflective. However, the analysis of deep choroidal blood flow was challenging in normal eyes, as the correlation signal was not generated in the presence of an intact RPE, given its reflectivity and the low flow rate at the choroidal level. In normal eyes with intact retinal pigment epithelium (RPE), the vascular lamina is not visualized with OCT-A, due to the hyper-reflective properties of the pigmented epithelium and the low flow velocity in the choroidal vascularization [23, 24]. However, it is possible to visualize the vascularization at the level large vessels of the Sattler and Haller layers in cases of geographic atrophy for “window effect” [25]. For this reason, OCT-A images acquired in cases of geographical atrophy have been included in our series. Further studies are needed to better define the best configuration for analyzing these choroidal layers with OCT-A.

Nerves and melanocytes, are not visualized on OCT and OCT-A which thus far can only provide good images of blood vessels in Sattler's and Haller's layers in eyes with atrophy of the retinal pigment epithelium. However, indirect correlations between histopathological images of the choroid and its innervation are still possible, combining the information provided by the two different modalities. OCT provided qualitative and quantitative analysis of the blood flow and choroidal thickness, which it is related to the neural activity on vessels, smooth muscle cells or melanocytes present in the intervascular spaces, as shown also by our histochemical images. Different studies proved that choroidal thickness undergoes several diurnal fluctuations [26–28]. For instance, choroidal thickness changed in response to blurred images, bringing the fovea to the ideal focus position. Recently, Read et al. [29] showed through ocular biometry similar changes in choroidal thickness in humans in response to short-term monolateral defocus.

Evaluation of choroidal thickness changes plays an important role in diseases such as central serous chorioretinopathy or chorioretinitis such as Vogt-Koyanagi-Harada syndrome [30–32]. A pronounced loss of choroidal thickness may occur with age, leading to a progressive choroidal atrophy similar to that of high myopia. These findings were so relevant to lead to a new pathological entity called “age-related choroidal atrophy”, characterized by widespread choroidal thinning with more or less preserved visual acuity [33]. Furthermore, age progression may induce alterations in choroidal adrenergic innervation leading to degenerative conditions. For this reason, choroidal thickness can be an indirect sign not only of vascular changes, but also of variation of vascular control dependent on adrenergic fibers, given their extent and distribution. However, Sogawa et al. [34] in studying the relationship between choroidal thickness and the relative blood flow, were not able to identify significant correlations.

To date, the relationship between choroidal thickness and blood flow has not been completely revealed. Moreover, different values of intraocular pressure can affect the choroidal thickness and choroidal blood flow, both reduced in case of elevated intraocular pressure. In this sense, adrenergic intervascular fibers and choroidal melanocytes could play an important role in the regulation of choroidal blood flow in response to changes in intraocular pressure.

## Conclusions

New developments in OCT technology have dramatically improved morphological and functional understanding of chorioretinal tissues. Emerging possibilities such as OCT en-face and OCT angiography can have a pivotal role in ophthalmological research and clinical practice. However, there are still fundamental aspects that need to be improved, to date. For example, more sophisticated algorithms would allow automatic measurements not only of the total thickness of the choroid, but also a differentiation of each individual layer. The state of choroidal innervation cannot be assessed on the OCT examination, nor other components featuring a regulatory relationship with choroidal vasculature, such as intervascular nerve fibers or choroidal melanocytes, can be identified. Therefore, modifications of structures such as vessels and melanocytes, modulated by adrenergic fibers, which may have a role in the control of choroidal blood flow and choroidal thickness, are not visible. The only characteristic that we are able to evaluate is represented by choroidal thickness, which can only be an indirect sign of adrenergic fibers and melanocytes activity (dependent on adrenergic innervation), as well as of intervascular smooth muscle cells [35].

OCT-A does not currently allow an exact evaluation of the dynamic changes of choroidal blood flow. The significant system regulating choroidal blood flow, characterized by adrenergic innervation and melanocytes should be taken into consideration. Therefore, histopathologic analysis of these structures may provide insights for future interpretation of choroidal OCT angiography findings, with broader possible applications. For now, OCT angiography plays a useful role in the clinical evaluation of macular disorders, leading to new parameters for clinical management. The choroid remains an extremely dynamic structure, deserving to be analyzed in vivo, in real time and in all the aspects that contribute to its dynamism.

## Data Availability

Data sharing is not applicable to this article as no datasets were generated or analyzed during the current study.

## Abbreviations

OCT: Optical coherence tomography
EDI-OCT: Enhanced depth imaging OCT
SS-OCT: Swept source OCT
OCT-A: OCT angiography
IF: Induced fluorescence
